# The *Qanuilirpitaa?* 2017 Nunavik Health Survey: design, methods, and lessons learned

**DOI:** 10.17269/s41997-023-00846-6

**Published:** 2024-01-17

**Authors:** Pierre Ayotte, Susie Gagnon, Mylène Riva, Gina Muckle, Denis Hamel, Richard E. Bélanger, Christopher Fletcher, Christopher Furgal, Aimée Dawson, Chantal Galarneau, Mélanie Lemire, Marie-Josée Gauthier, Elena Labranche, Lucy Grey, Marie Rochette, Françoise Bouchard

**Affiliations:** 1 Département de médecine sociale et préventive, Faculté de médecine, Université Laval, Québec, QC Canada; 2 Axe en santé des populations et pratiques optimales en santé, CRCHU de Québec – Université Laval, Québec, QC Canada; 3 Direction de la santé environnementale, au travail et de la toxicologie, Institut national de santé publique du Québec, Québec, QC Canada; 4 Bureau d’information et d’études en santé des populations, Institut national de santé publique du Québec, Québec, QC Canada; 5 Department of Geography, McGill University, Montreal, QC Canada; 6 École de psychologie, Université Laval, Québec, QC Canada; 7 Département de pédiatrie, Faculté de médecine, Université Laval, Québec, QC Canada; 8 Indigenous Environmental Studies and Sciences Program, Trent University, Peterborough, ON Canada; 9 Faculté de médecine dentaire, Université Laval, Québec, QC Canada; 10 Direction du développement des individus et des communautés, Institut national de santé publique du Québec, Québec, QC Canada; 11 Institut de biologie intégrative et des systèmes (IBIS), Université Laval, Québec, QC Canada; 12 Public Health Department, Nunavik Regional Board of Health and Social Services, Kuujjuaq, QC Canada; 13 Inuit Values and Practices, Nunavik Regional Board of Health and Social Services, Kuujjuaq, QC Canada

**Keywords:** Nunavik, Inuit, Health survey, Community health, Participatory research, Nunavik, Inuit, enquête de santé, santé communautaire, recherche participative

## Abstract

**Objective:**

To depict the design, methods, sociodemographic characteristics of the population, and lessons learned during the *Qanuilirpitaa?* 2017 Nunavik Inuit Health Survey, the third major health survey to be conducted among youth and adults residing in Nunavik (Northern Quebec, Canada).

**Methods:**

*Qanuilirpitaa?* 2017 is a cross-sectional survey that served to update information regarding various aspects of physical health, mental health, and general well-being of Nunavimmiut. The survey was guided by the ethics principles of Ownership, Control, Access, and Possession (OCAP®) (https://fnigc.ca/ocap). Questionnaires and clinical tests were administered to residents from the 14 coastal communities onboard the Canadian Coast Guard Ship *Amundsen* during late summer and early fall 2017. As part of the community component of the survey, qualitative interviews were performed with key respondents, and services and resources supporting health and well-being in the 14 communities were inventoried and characterized.

**Results:**

A total of 1326 Nunavimmiut aged 16 and over participated in the survey. Despite difficulties encountered with the recruitment of participants, co-interpretation sessions with Inuit partners revealed that the survey had succeeded in capturing cultural, socio-economic, and lifestyle characteristics of Nunavimmiut. In all, 20 thematic reports have been published covering various aspects of health and well-being of Nunavimmiut. Regional and local reports pertaining to the community component were produced. More in-depth analyses have ensued, and results are presented in articles published in this CJPH supplement issue.

**Conclusion:**

Information from this survey is being used to update health services and programs in the region and for the development of health policies and public health interventions to tackle key health-related issues faced by Nunavimmiut. Drawing lessons from challenges and successes encountered in *Qanuilirpitaa?* 2017, this survey paved the way to the upcoming Inuit-led *Qanuippitaa?* National Inuit Health Survey to be conducted every 5 years throughout Inuit Nunangat.

**Supplementary Information:**

The online version contains supplementary material available at 10.17269/s41997-023-00846-6.

## Résumé

**Objectif:**

Décrire la conception, les méthodes, les caractéristiques sociodémographiques de la population et les leçons tirées de l’Enquête sur la santé des Inuits du Nunavik *Qanuilirpitaa?* 2017, la troisième grande enquête de santé menée auprès de jeunes et d’adultes résidant au Nunavik (Nord du Québec, Canada).

**Méthodes:**

*Qanuilirpitaa?* 2017 est une enquête transversale qui a permis la mise à jour des informations concernant divers aspects de la santé physique, de la santé mentale et du bien-être général des Nunavimmiut. L’enquête a été guidée par les principes de propriété, de contrôle, d’accès et de possession (OCAP®) (https://fnigc.ca/ocap). Des questionnaires et des tests cliniques ont été administrés à des résidents des 14 communautés côtières, à bord du navire Amundsen de la Garde côtière canadienne, à la fin de l’été et au début de l’automne 2017. Des entrevues qualitatives ont également été réalisées avec des répondants clé dans le cadre de la composante communautaire.

**Résultats:**

Au total, 1 326 Nunavimmiut âgés de 16 ans et plus ont été recrutés. Malgré les difficultés rencontrées lors du recrutement des participants, les rencontres de co-interprétation ont permis de vérifier que l’enquête avait réussi à capturer les caractéristiques culturelles, socioéconomiques et les habitudes de vie de la population. Au total, 20 rapports thématiques ont été publiés, lesquels couvrent différents aspects de la santé et du bien-être des Nunavimmiut. Un rapport régional et des rapports locaux liés à la composante communautaire ont également été produits. Des analyses plus approfondies ont également été réalisées, dont les résultats sont présentés dans les articles publiés dans ce numéro supplémentaire de la Revue canadienne de santé publique.

**Conclusion:**

Les informations issues de cette enquête sont utilisées pour la mise à jour des services de santé dans la région et pour le développement de politiques de santé et d’interventions de santé publique, ayant pour cibles les principaux défis auxquels les Nunavimmiut sont confrontés. Grâce aux leçons apprises lors de sa réalisation, cette enquête a pavé la voie aux prochaines enquêtes *Qanuippitaa ?* qui seront menées tous les 5 ans dans tout l’Inuit Nunangat.

## Introduction

Assessing the health status of Inuit living in Nunavik requires careful attention considering the unique historical, cultural, social, and geographic contexts of the region. In this article, we describe the design and methodological components of the *Qanuilirpitaa?* 2017 Nunavik Health Survey (Q2017) and conclude reflections on the lessons we have learned.

Inuit have faced considerable challenges since the arrival of traders, missionaries, government, and other agents who brought major social, economic, and cultural changes throughout the Inuit Nunangat. Changes in Inuit lives accelerated in the mid-twentieth century, which saw the creation of the permanent communities and marked the incorporation of Inuit as citizens within the Canadian state. Surveys played an important role in describing the health of Inuit in the post-war period, shaping and justifying arctic policy and program development—the effects of which are expressed in particular patterns of health and illness today. Health surveys for Inuit have thus been intricately linked to social transformation and new arrangements of people and places. With the conclusion of land-claims negotiations in the mid-1970s, Inuit in Nunavik began building institutional capacity and partnerships to assess the health status of the population and deliver programs and services tailored to their needs.

Three health surveys were previously conducted in Nunavik: the Plasanouq survey in 1982–1984, the Santé Québec survey in 1992, and the *Qanuippitaa?* health survey in 2004. In February 2014, the Board of Directors of the Nunavik Regional Board of Health and Social Services (NRBHSS), composed of representatives from the region’s 14 communities and from its two health centres, unanimously adopted a resolution to conduct a new health survey focusing on three components: adult health, youth health, and community health. The Q2017 survey represents an important step on the path to Inuit-led and -controlled health research. The design and methods reflect the complex logistical challenges of conducting health surveys in the remote communities of Nunavik, using methodologically sound epidemiological survey research and survey tools that are culturally and linguistically appropriate.

The overall aim of Q2017 was to establish a current portrait of the health status of Nunavimmiut aged 16 and older, and to identify health priorities for adapting services and programs to respond to current needs of the population. Additional goals were to assess temporal trends in the health status of this population undergoing rapid and significant lifestyle changes, and to gain more understanding of the interplay between factors which affect the health and well-being of Nunavimmiut. Furthermore, Q2017 included a “community component,” which focused on the perspective and the experience of people in their home communities.

## Methods

### Participatory approach

#### Partners and principles

The *Qanuilirpitaa?* health survey was forged through partnerships among all major Nunavik organizations (NRBHSS, Makivvik Corporation, Kativik Regional Government (KRG), Kativik Ilisarniliriniq (KI), Avataq Cultural Institute, Qarjuit Youth Council), the two health centres (Inuulitsivik and Ungava Tulattavik), representatives of the community mayors, the *Institut national de santé publique du Québec* (INSPQ), and academic researchers from Université Laval, McGill University, and Trent University. Q2017 was guided by the principles and values of Ownership, Control, Access, and Possession (OCAP®) and privileged Inuit rights and interests (First Nations Information Governance Centre, 2016). OCAP® principles were formalized in a data management policy that provides clear direction for the management of, access to, and use of the survey data. Under the agreement for this survey, Nunavik institutions own all data and biological samples, and the intellectual property of the survey results is shared between the region and the researchers. Each researcher is required to sign the confidentiality agreements to respect the security of the information and the protection of personal information.

#### Governance

Governance of the survey was overseen by a steering committee composed of representatives of the major Nunavik organizations listed above, the health centres, and mayors representing the communities. Representatives of the INSPQ and *Centre de recherche du CHU de* *Québec*-*Université Laval* were also members of the Steering Committee. The NRBHSS chaired the Steering Committee and was responsible for overall survey conduct.

**Fig. 1:**
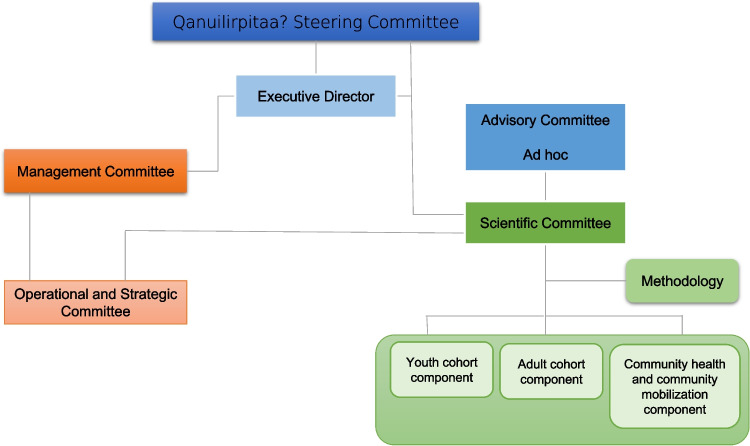
Governance structure of the *Qanuilirpitaa*? 2017 Health Survey

Three partners worked on planning to conduct the survey: NRBHSS, INSPQ, and the *Centre de recherche du CHU de Québec-Université Laval*. In addition, the NRBHSS was responsible for communicating the survey results through publications on their website (see the Knowledge translation and communication section). The INSPQ was mandated with designing the sampling frame and selecting appropriate statistical analyses for the survey. The Institute also ensured overall co-ordination and administrative management. A team of academic researchers from different universities selected and adapted the clinical tests and questionnaires. That group was in charge of conducting the medical chart review that addresses the different themes of the survey. Researchers oversaw the statistical analyses and co-interpreted the data together with Inuit colleagues. The governance structure also included different committees and subcommittees composed of individuals from the NRBHSS, representatives from Inuit communities and organizations, principal academic investigators, and the INSPQ (Fig. 1). This decision-making structure was intended to allow Nunavimmiut to actively participate in and lead or co-lead all phases of the health survey.

#### Consultations and identification of themes/topics

An extensive consultation took place in Kuujjuaq on January 27 and 28, 2015, to discuss with key informants from the communities and organizations in Nunavik on the themes and topics to prioritize in Q2017. Other consultations were subsequently held in 2015 and 2016 that led to the selection of the themes that are listed in Table 1, along with the targeted cohorts—youth (16 to 30 years old) and/or adults (31 and older).

**Table 1:** Themes selected for the *Qanuilirpitaa*? 2017 Health Survey

Theme	Target cohort*
Sociodemographic information (including education, income, employment)	Youth and adults
Subjective rating of overall mental and physical health (quality of life, self-rated health, satisfaction in life)	Youth and adults
Mental health and well-being (psychological distress, stress, suicide, traumas, historical loss, community attitudes toward mental health)	Youth and adults
Resilience and self-esteem	Youth and adults
Social support	Youth and adults
Victimization	
- Crime against property - Physical and sexual abuse - Childhood trauma - Elder victimization - Bullying	Youth and adults Youth (18–30 years) and adults Youth (18–30 years) and adults Adults 55 and over Youth
Sexual health (sexual attraction, perceived benefits of childbearing, risky sexual behaviours in association with sexually transmitted infections)	Youth
Reproductive history	Youth and adults (men and women)
Adolescent pregnancy (sociocultural factors influencing teenage pregnancy)	16–20 years
Justice	Youth and adults
Substance use and dependence, gambling	Youth and adults
Use of social media	Youth and adults
Family (family relations and support, cohesion, family traumas (stressors), residential school, foster care)	Youth and adults
Sociocultural determinants of health (incl. cultural identity, community involvement, equity and discrimination, social integration and exclusion, spirituality)	Youth and adults
Housing and homelessness	Youth and adults
Cardiometabolic health (obesity, diabetes, hypertension, dyslipidemia, fatty liver, and fibrosis)	Youth and adults
Acute gastroenteritis infection and *Cryptosporidium* spp.	Youth and adults
Zoonosis (rabies infection and risk of bites, *Toxoplasma gondii* and *Trichinella* spp.)	Youth and adults
Sexually transmitted infections	Youth and adults
*Helicobacter pylori*	Youth and adults
Oral health	Youth and adults
Respiratory health	Youth and adults
Men’s health	Men from both cohorts
Women’s health	Women from both cohorts
Nutrition and anemia, food security, chemical contaminants	Youth and adults
Hunting and fishing	Youth and adults

*Unless otherwise specified, youth correspond to the 16 to 30 age group and adults to those aged 31 and older

#### Ethics and consent

Q2017 received ethics approval by the *Comité d’éthique de la recherche du Centre Hospitalier Universitaire de Québec-Université Laval *(#2016–2499 and #2016–2499-21) on ethical aspects of the project. In each community, potential participants were identified and contacted by the recruitment team for their possible participation in the survey. Inuktitut translation was provided as needed. During this visit, individuals were given a brief description of the survey and invited to watch a short video (in Inuktitut, English, or French). These were followed up with consent forms and each participant was informed on clinical and laboratory procedures.

### Sampling strategy

The target population for the survey consisted of all permanent residents of Nunavik, Inuit and non-Inuit, aged 16 years and over on the register of the beneficiaries of the James Bay and Northern Quebec Agreement (JBNQA) provided to the research team by Makivvik Corporation in the spring of 2017. Individuals living in institutional settings (e.g., care homes, healthcare facilities, or prisons), people suffering from tuberculosis, those unable to come on the ship for health reasons, and members of the survey staff were excluded.

Non-proportional stratified sampling was used with strata defined according to sex, age group (16–19 years old, 20–30 years old, and 31 years old and over), and community of residence. For the 16–30 age group, individuals were selected within each stratum using simple random sampling without replacement. For the older group, the basic sample consisted of members of the 2004 *Qanuippitaa?* cohort (Chateau-Degat et al., 2010) plus an additional sample selected using simple random sampling without replacement. This approach allowed for the longitudinal monitoring of the 2004 cohort as well as the production of estimates for the older age group in 2017.

Weighting of survey data was achieved in three steps which are described in detail in the methodological report of the survey (Hamel et al., 2020). First, an initial weight was attributed that corresponds to the inverse of the probability of selection, which is directly dependent on the sampling design described above. Second, a non-response adjustment of the initial weights was performed in an attempt to minimize the risk of selection bias resulting from the large non-response rate in the survey. Unfortunately, the number of variables available in the original sampling list (JBNQA beneficiaries) was limited and only allowed adjustments based on variables similar to those used for stratification purposes: sex, age group (16–30 years, 31–45 years, and 46 years and over), and community of residence. Third, weights were adjusted to take into account another form of non-response—“no-shows”—i.e., participants who for any reason missed their appointment after agreeing to participate. Annual income, sex, and community of residence were used in this last weight adjustment.

### Logistics

As for the *Qanuippitaa?* survey in 2004, data collection for Q2017 was conducted by the survey staff traveling from community to community onboard the Canadian Coast Guard Ship Amundsen, from August 19 to October 5, 2017*.*

#### Promotional campaign

In collaboration with the mayors of the 14 communities, the NRBHSS initiated an information and promotional campaign targeting Nunavimmiut and regional organizations. Several communication tools were used to meet the objectives laid out in the communication plan. A Facebook page was created to allow easy access to online videos produced about the survey. Scripted radio messages were recorded and aired on regional and local radio stations prior to the survey. Radio messages were delivered on local FM radio stations in each community in the days leading up to the ship’s arrival. Posters displaying the Q2017 logo and the ship’s schedule were delivered to all communities several weeks in advance.

#### Training of staff

Survey staff aboard the ship included the mission head, the clinical coordinator, 16 interviewers (eight Inuit and eight non-Inuit), five nurses, the laboratory coordinator, two laboratory technicians, two respiratory therapists, two dentists, one dental assistant, one dental hygienist, and one computer technician. A ground crew composed of 15 people was in charge of recruiting participants ahead of the ship reaching each of the 14 communities.

Interviewers received two and a half days of training on how to conduct interviews using a standardized approach, and they reviewed case studies involving potentially difficult situations. A nutrition specialist provided specific information on the food frequency questionnaire section. The interviewers were asked to fill out the questionnaires in order to familiarize themselves with the way in which the questions were formulated and the data were being entered.

Several health professionals were trained for the clinical part of the survey. Nurses were responsible for collecting biological samples and performing certain clinical measurements (see below). Training was provided by the clinical coordinator and a Canadian Coast Guard nurse, who was present on the ship during the survey. Two respiratory therapists in charge of the lung health assessment were given a 1-day training session on the spirometry procedure at the McGill University Health Centre (MUHC). Dentists and their assistants who were involved in the oral health component received a 4-day training covering clinical measures, material and equipment, and data collection tool. Part of the training and calibration of examiners took place in a real setting with volunteers undergoing the oral clinical examination as planned for the survey.

### Data collection

#### Questionnaires

The questionnaires for each theme were developed by researchers accompanied by Inuit co-leaders. The questions were selected to (1) enable comparisons with the 2004 health survey; (2) allow comparisons with other Canadians; and (3) provide useful information to the region for advocacy and policy programming. Notwithstanding the comparability needs between surveys, questions that were not well understood or deemed not useful in 2004 were not included in the 2017 questionnaires; they were replaced by questions addressing the same topics that were previously tested and validated among Inuit or other Indigenous People. Questions were grouped in different blocks that addressed the various themes covered in the survey (Table S1, Supplementary Material).

Questionnaires were written in English, translated into Inuktitut by an Inuk employed at the NRBHSS, and then verified by another translator at the NRBHSS. The questionnaires were pre-tested in November 2016 with people from the community of Inukjuak to verify comprehension of questions, sensitivity issues, laptop and software programming challenges, and total duration required for questionnaire administration. The pre-test revealed that the time required to complete the interview was too long and several questions were subsequently eliminated, in cooperation with research team members. Questions that proved difficult to understand during the pre-test were either modified or replaced.

Whereas questions in blocks 1 to 4 were administered onboard the ship, individuals first answered the sociodemographic portion of the questionnaire (block 5) at the recruitment office in each community. The recruiter then scheduled a morning or afternoon appointment to visit the research clinic on board the ship and provided the participant a container and instructions for stool collection. Participants invited to the morning session were asked to arrive at their appointment after fasting for a minimum of 12 h.

#### Clinical and laboratory data

##### Anthropometry

Anthropometric measures were collected by nurses or trained interviewers with a tape measure (cm) during the clinical session. Height was measured while the participant was standing on a hard surface without shoes. Waist circumference was measured, after expiration, at the mid-point between the iliac crest and the last loose rib, twice, and the mean of the two measurements was used (Hamel et al., 2020). Weight and body composition were measured on a bioelectrical impedance analysis instrument (InBody 570, InBody Canada, Ottawa, ON) while the participant was standing barefoot on the plate and holding hand electrodes. More details can be found in the thematic report on cardiometabolic health (Allaire et al. 2021a).

##### Blood pressure

Nurses used a Welch Allyn ProBP 2400 Digital Blood Pressure Device to measure blood pressure and pulse of participants. Blood pressure was measured according to the 2005 Canadian Hypertension Education Program (Hemmelgarn et al., 2005). Three measurements were taken from the same arm, after the participant rested for 5 min in the seated position with back support. The first reading was discarded and the latter two averaged to yield the participant’s blood pressure. More details regarding blood pressure measurements can be found in the thematic report on cardiometabolic health (Allaire et al., 2021a) and in Allaire et al. (this issue).

##### Pulmonary function testing

Spirometry is the most reproducible and objective measurement of airflow limitation (GOLD, 2017). Each participant was asked to perform a spirometry test following the protocol elaborated for the CanCOLD study (Bourbeau et al., 2012). Exclusion criteria were the following: being more than 27 weeks pregnant; recent heart attack (past 3 months); major chest or abdomen surgery (past 3 months); eye surgery (past 6 weeks); current medication for tuberculosis; tracheostomy; extreme difficulty breathing at rest; acute or chronic condition preventing the administration of the test (e.g., persistent cough). More details can be found in the thematic report on respiratory health (Robert et al., 2020) and in Robert et al. (this issue).

##### Oral health

A dentist examiner performed an oral health examination during which the following information was collected: (1) dentate status; (2) utilization of removable prostheses; (3) dental trauma; (4) gingival status; (5) debris and calculus assessment; (6) dental caries and associated conditions (ICDAS II); (7) clinical consequences of untreated dental caries. Participants with certain heart valve diseases were excluded from the gingival index, in accordance with NHANES 2011–2012 guidelines (NHANES, 2013). More details can be found in the thematic report on oral health (Galarneau et al., 2020) and the methodological report of the survey (Hamel et al., 2020).

##### Biological sample collection and analyses

Different biological samples were obtained from participants on board the ship. Nurses collected blood samples from an antecubital vein (67 mL) and oropharyngeal swabs, whereas participants collected their own urine samples (on the ship) and stool samples (at home). Women aged 16 to 30 collected their own vaginal swabs (on the ship). Samples were kept on ice and transported to laboratory on board the ship where they were processed, aliquoted, and stored in − 80 °C freezers. Complete blood count was performed on an aliquot of fresh whole blood using a DxH 500 hematology analyzer (Beckman Coulter, Mississauga, ON). Table S2 (Supplementary Material) lists the laboratory analyses performed on the different biological samples, the targeted cohorts, and the rationale for inclusion in the survey. A detailed description of the procedures for sampling, processing, and storing biological samples and laboratory analyses is available upon request to the INSPQ.

#### Medical chart review

A nurse visited the CLSC in each community, where she had access to the records and was provided with office space. As for the previous survey conducted in 2004, information was collected on different pathologies (cardiovascular diseases, cancer, metabolic disorders including diabetes, neurological affections, musculoskeletal problems) diagnosed using the International Classification of Diseases (ICD-11). New information on respiratory health and *Helicobacter pylori* infection was also recorded. In addition, information was collected on medication usage at the time of the survey.

### Community health component

At an international Inuit health survey workshop held in Kuujjuaq in 2012, participants formulated the need to expand the scope of the next circumpolar public health surveys to include an exploration of health as perceived and lived by Inuit residents. In 2014, building on this consensus, the NRBHSS Board of Directors decided to go forward with the *Qanuilirpitaa?* survey that would include youth and adult cohorts, and a “community component” that would consider this new perspective. The goal of the community component was to describe health from the perspective and experience of people in their home communities.

The activities that led to the realization of the community component of Q2017 are described in greater depth in Fletcher et al. (this issue). The community component had many objectives related to describing Inuit cultural concepts of health and well-being in relation to health determinants and community living; improving the understanding of how conditions and resources in communities contribute to the health of people; and providing data and information to develop community action plans and interventions to respond to the health and well-being needs of the residents of Nunavik. Data collection approaches were diverse, and included 1.5-day workshops in two communities, in-depth interviews with key informants in each of the communities, and a mapping exercise that identified and characterized community resources supporting health and well-being. Outputs were validated with community members and with the NRBHSS.

The community component developed a new model of health and well-being, the *Ilusirsusiarniq, Qanuinngisiarniq, and Inuuqatigiitsianiq* (IQI) model, which is presented in Fletcher et al. (this issue). Portraits for each of the 14 communities were also prepared that focus on community strengths with respect to eight social determinants of health that support people to be healthy according to the IQI model. These portraits can be accessed through the NRBHSS website (https://nrbhss.ca/en/nrbhss/public-health/nunavik-health-surveys/qanuilirpitaa-2017). The community component was designed to provide information to a community health mobilization program which is currently underway after significant delays caused by the COVID-19 pandemic.

### Data management

The INSPQ has been entrusted with managing and storing the data and biological samples. Access to the data is provided on approval by the Data Management Committee (DMC). The DMC is composed of members from major Nunavik organizations and oversees the management of the survey data and biological samples. Before granting access, analysis plans are reviewed to ensure the questions addressed are of value for the region. Abstracts and manuscripts produced by researchers are submitted to the DMC for co-interpretation by the members and approval by the committee. The data management policy is available upon request to the NRBHSS.

### Knowledge translation and communication

#### Participant results

A health passport containing key clinical results was given to each participant prior to their leaving the ship (shown in part in Figure S1, Supplementary Material). All clinical data were shared with the CLSC of the participant to be included in their medical file. Health conditions requiring immediate attention (i.e., severe anemia) were immediately referred to the clinical services of the region.

#### Communication of survey results

A total of 20 thematic reports were produced on the different themes covered by the survey (Table 1). Each thematic report is accompanied by a summary in plain language and an infographic describing key findings and related public health information (in Inuktitut, English, and French). Fourteen community portraits and a report on the IQI model of health and well-being were also prepared. These documents, published between 2020 and 2023, are available on the NRBHSS website (http://nrbhss.ca/en/nrbhss/public-health/nunavik-health-surveys/qanuilirpitaa-2017).

Since the publication of the results, the NRBHSS has been actively promoting the *Qanuilirpitaa?* 2017 survey results to the Nunavik organizations and associations so they can be utilized to inform Nunavik services, programs, and policies. The NRBHSS had also planned to visit Nunavik communities to present the main survey results (especially pertaining to the community component) and discuss opportunities for action with residents and representatives. These activities which have been delayed due to the COVID-19 pandemic have started since the fall of 2022 and are currently underway.

## Results

A total of 1326 people from the 14 communities of Nunavik took part in the survey. To achieve this number, recruitment teams met with 1661 eligible people who had agreed to participate in the survey. Difficulties encountered at the time of recruitment meant that the target of 2000 participants was not reached. Globally, the participation rate was 37% among sampled individuals. Out of those successfully contacted, 80% participated in the survey.

### Sociodemographic characteristics of the population sample

**Fig. 2:**
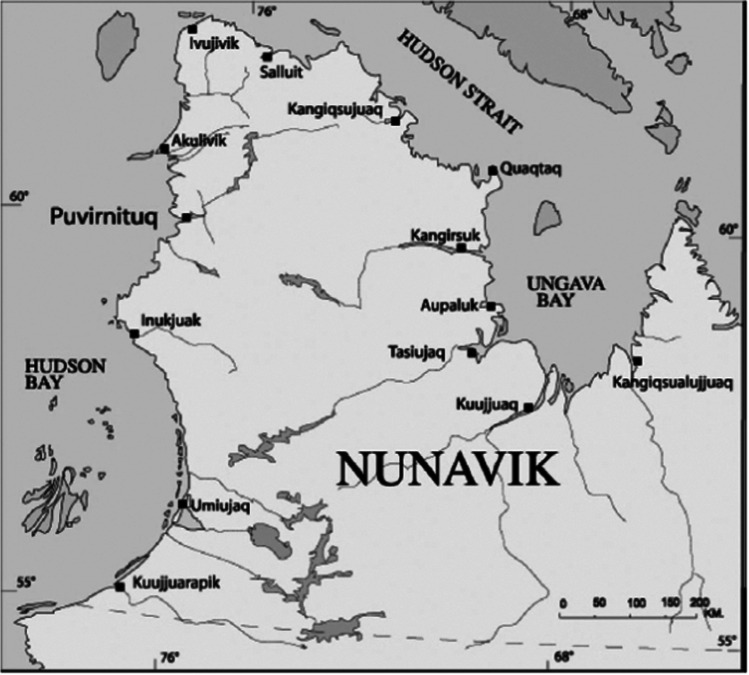
Map of Nunavik. Source: Makivvik Corporation

Table 2 describes the survey sample according to age group, sex, and subregion defined for administrative purposes and inclusion in the Circumpolar Inuit Health in Transition cohort study initiated in 2004. The geographical location of the 14 Nunavik communities is shown in Fig. 2.

**Table 2:** Final distribution of the sample of participants by age, sex, and administrative subregions, *Qanuilirpitaa*? 2017 Health Survey

Cohort	16–30 years		31 years and over				Total		
Subregion*	Women	Men	Women		Men		Women	Men	Total
			All	2004 cohort**	All	2004 cohort**			
Hudson Coast	200	101	220	98	133	52	420	234	654
Ungava Coast	194	79	259	98	140	55	453	219	672
Total	394	180	479	196	273	107	873	453	1326

*Hudson Coast: Kuujjuarapik, Umiujaq, Inukjuak, Puvirnituq, Akulivik, Ivujivik, and Salluit; Ungava Coast: Kangiqsujuaq, Quaqtaq, Kangirsuk, Aupaluk, Tasiujaq, Kangiqsualujjuaq, and Kuujjuaq

**Participants in the *Qanuippitaa?* 2004 survey enrolled in the Circumpolar Inuit Health in Transition cohort study (Chateau-Degat et al., 2010)

The population sample was equally distributed between the Hudson Coast and the Ungava Coast regions. One thousand three hundred individuals self-identified as Inuit, 16 as Caucasian, and ten as “other.” Nearly twice as many women as men participated in the survey. The youth cohort (16–30 years) comprised 574 individuals and the adult cohort (31 +) 752 individuals. Three hundred and three Nunavimmiut who were enrolled in the Circumpolar Inuit Health in Transition cohort study in 2004 (Chateau-Degat et al., 2010) were included in the adult cohort in 2017. A longitudinal assessment will therefore be possible for some health outcomes and determinants.

## Discussion

*Qanuilirpitaa?* 2017 was the third major health survey carried out in Nunavik, following *Qanuippitaa?* in 2004 and the Santé Québec Inuit Health survey in 1992. Health surveys in Nunavik have progressively moved from a public health monitoring approach to an engaged partnership between regional organizations, academic researchers, and the Institut national de santé publique du Québec (INSPQ), building on the foundations of a successful collaborative relationship established during the *Qanuippitaa?* 2004 survey (Fletcher, 2017). The Q2017 survey went beyond traditional survey approaches as it became more responsive to Inuit needs and thus involved a high level of active participation of the communities at all phases of the survey, with the objective of making the survey increasingly relevant and useful for Nunavimmiut, as well as strengthening autonomy, local capacities, and empowerment, in respect of Inuit self-determination in decision-making. Q2017 paved the way for the next surveys to be held every 5 years throughout Inuit Nunangat—*Qanuippitaa?* National Inuit Health Survey—which will be conducted entirely under Inuit leadership. It is therefore important to identify what worked well and what were the main challenges of Q2017 to inform the upcoming *Qanuippitaa?* surveys.

Health surveys mainly serve to update information on the health and well-being of Nunavimmiut, allowing advocacy for improved health care services and programs to meet the current needs of the population. Services and programs must also be in line with the conception of health and well-being of the population. The IQI health model developed in the framework of Q2017 (see Fletcher et al., this issue) will help in designing communication strategies and will inform future *Qanuippitaa?* health surveys to be conducted in the region.

Some key findings of the survey regarding aspects of physical health are further explored in articles published in this CJPH special issue: the high prevalence of hypertension and respiratory symptoms, especially in youth (Allaire et al., Robert et al., this issue), and the “moderate” public health problem of anemia among women of reproductive age, despite an apparent improvement since the last survey in 2004 (Lavoie et al., this issue). Other articles focus on the sociocultural determinants of mental health (Poliakova et al., this issue) and substance use (Courtemanche et al., this issue).

Furthermore, the survey provided an opportunity for participants to access some health services that are not readily available in the region. Screening for colorectal cancer and for sexually transmitted and blood-borne infections (STBBIs), respiratory and cardiometabolic health assessments, and an extensive oral health exam were offered to Q2017 participants.

### Representativeness, participation rate, and limitations

Despite the strategies to engage and mobilize the population (see sections reporting on Logistics and Promotional campaign), the recruitment of participants was difficult and yielded a relatively low response rate (31% for people aged 16 to 30 years and 41% for people aged 31 and over) (Hamel et al., 2020). This was largely due to the higher than anticipated non-contact rate (38% for people aged 16 to 30 and 29% for 31 and over). A variety of factors explain this situation, the high mobility of people in Nunavik topmost among them: individuals were away working for several days, away for study, or spending time on the land for harvesting. Difficulties in reaching individuals identified in the initial sample led to an overuse of the replacement sample. When paired with the no-shows, this response rate increases the risk of selection bias, i.e., that the population sample may not be representative of the Nunavik population aged 16 and over (Hamel et al., 2020). Unfortunately, the direction in which estimates may be biased cannot be predicted, as both individuals who are well-off and individuals who are harder to reach may have been underrepresented in the final sample. The necessity to be able to travel on a small boat to board the ship where data collection occurred likely prevented the participation of some individuals with physical mobility issues. Conducting data collection directly in the communities, avoiding the summer/early fall period (high season for land-based activities), and spending more time to recruit individuals could reduce the probability of selection bias in future *Qanuippitaa?* surveys.

Discussions of survey results during co-interpretation sessions indicated that the *Qanuilirpitaa?* 2017 survey had succeeded in capturing the diversity of socio-economic status, food security status, dietary and lifestyle habits, and Inuit culture adherence encountered in the Nunavik population (data available in the following thematic reports: Allaire et al., 2021b; Furgal et al., 2021; Muckle et al., 2020; Riva et al., 2020). For example, Aker et al. (this issue) report on the different food consumption profiles encountered in the Nunavik population. High consumers of both country and market foods, dominant market food consumers, dominant country food consumers, and low consumers of either country or market foods are described. These profiles are in agreement with the perception of Inuit collaborators and nutritionists knowledgeable of dietary habits in Nunavik, thereby providing further confidence in the representativeness of our population sample.

### Lessons learned: informing future health surveys in Nunavik

#### Participatory approach takes time, and then more time

One of the main challenges of Q2017 was defining a health survey, its processes, tools, governance, and knowledge mobilization strategies that properly reflected Inuit conceptions and experiences of health and well-being, while also meeting public health needs for surveillance indicators that are useful, methodologically robust, temporally comparable, and scientifically rigorous. Reconciling the differing agendas and needs of the various partners to come to mutual understanding, and building the foundations for successful, trustworthy, and mutually beneficial partnerships take time and commitment (Lyons, 2011).

Being creative and alternating meeting places between Nunavik and the south to accommodate people are essential. Setting realistic expectations; being open-minded; showing flexibility, respect, and humility; and being able to adapt to different work rhythms are all significant personal abilities supporting success in this context. The first discussions regarding a new health survey occurred in 2012, the resolution to conduct Q2017 was adopted in 2014, and the survey was implemented in 2017, with data collection taking place in late summer-early fall of that year. Data analysis, co-interpretation of the results, and writing of thematic reports and other communication materials extended from 2018 to 2022 and were considerably slowed down because of the COVID-19 pandemic, fatigue following the intense data collection phase, and staff turnover. Publishing these documents was a prerequisite to additional data analyses, co-interpretation, and publication in scientific journals, such as those presented in this CJPH special issue. Local leaders and stakeholders, as well as the research team, had many other commitments outside the survey during the life of this project. Although they showed engaged leadership and commitment, sustaining the interest, motivation, and active participation over that many years represented a great challenge, particularly considering other urgent obligations and responsibilities as well as high staff turnover.

#### Scope of the survey and burden on participants

In order to cover the many themes included in Q2017, participants were asked to answer a lengthy questionnaire and participate in a clinical session during which anthropometric measurements were taken and biological samples were obtained, in addition to submitting to an oral examination and a lung function test. These study goals resulted in a significant time commitment by the participant, often exceeding 3 h. Selecting the tools and questions that were validated, while also adapted to the Inuit context, with the objective of keeping the survey duration at a reasonable and feasible time, proved to be a significant challenge. In the end, some questions had to be removed from the questionnaire; thus, some themes could not be explored in depth and the results could only offer a brief overview of some health issues. Still, the survey questionnaire was very extensive and generated a large amount of useful data. Future health surveys should be conducted more regularly (i.e., every 5 years) and should focus on a smaller number of themes, allowing for a more comprehensive understanding of certain topics while decreasing the burden on participants.

#### Cultural sensitivity and safety issues

Despite substantial effort, some questions remained poorly adapted and were assessed as lacking cultural sensitivity in how they were to be asked. It was not sufficient to simply adapt questions from the South to the North or only use validated questionnaires from other contexts; questions are more likely to make sense to Nunavimmiut if they are conceptualized by Inuit in Inuktitut. Some participants experienced confusion when confronted by double negatives in questions and answer choices (e.g., “not at all” as a proposed answer to the question “It does not take me long to recover from a stressful event”). The interviewers also noted that some concepts seemed to be poorly understood, such as “spiritual values,” “bullying,” and “discrimination.” It was suggested that providing definitions and examples for unclear questions or concepts would be helpful.

Some questions were perceived as being intrusive or discriminatory by a number of respondents. Conducting a health survey investigating highly complex and sensitive issues, in a context of social suffering and historical trauma, requires special attention to help and support participants and research staff. It was difficult for some participants, as well as for some interviewers, to re-live individual experiences of traumas, including physical, sexual, and emotional violence, through the stories of the participants. We had anticipated that some participants would experience distress after being asked sensitive questions in the psychosocial interview. A list of available resources was provided to the participants, but more support would have been valuable. Many interviewers experienced fatigue and depressive symptoms and worry for participants who were visibly distressed. In debriefing interviews, a group of interviewers mentioned the need for dedicated professionals available on the ship to support them when needed as well as to offer appropriate support for the participants. Other strategies to address these issues were identified by the interviewers, including individual and group counselling, availability of debriefing spaces, devoting part of the training to the recognition of some signs of research staff and participant fatigue and distress, and follow-up with participants afterwards to help them process and overcome the distress caused by revisiting their trauma. In addition to providing adequate support for survey participants, future surveys should plan for substantive emotional and psychological support resources for survey staff, particularly interviewers, at regular intervals after the survey is completed.

Although significant efforts were made to ensure cultural safety at every stage of the Q2017 survey, there is clearly room for improvement. “Cultural safety is an increasingly widespread principle in the evaluation of health systems’ capacity to respond to the needs of the Indigenous populations they serve. It is now recognized as going well beyond conventional intercultural approaches, emphasizing that true quality of care can only be achieved when users feel perfectly safe throughout the health care trajectory” (NRBHSS 2021). This concept, used to describe the relationship between a caregiver and a patient, needs to be more thoroughly integrated in health surveys conducted in Nunavik.

Cultural safety is proving effective at promoting a critical perspective and moral discourse on how peoples’ health is influenced by historical, political, and socio-economic circumstances (Gerlach, 2012). It involves a change of paradigm and requires integrating the experiential knowledge and cultural depth and means enabling the Inuit to self-determination (NRBHSS, 2021).

The pre-survey training is an area where the intercultural aspects such as history, values, and ways to communicate could have been better integrated, within a cultural safety approach. Moreover, cultural awareness workshops should be part of the training and Inuit must play an even more active role at every step of the survey.

Finally, the majority of Q2017 participants wished to be interviewed in Inuktitut, but the number of interviewers who could speak the language was insufficient to meet the demand. The default language for research by Inuit and for Inuit should be Inuktitut, although differences are notable across Inuit Nunangat; every participant should be able to answer the questionnaire in their preferred language.

## Conclusion

The *Qanuilirpitaa?* 2017 provided important information on the health and well-being of Nunavimmiut that will support actions to update services and programs in Nunavik. Moreover, key information on how Inuit perceive their own health and that of their community was generated, paving the way for the next health surveys to be conducted in the region. Jointly with Inuit Tapiriit Kanatami, the four regions of Inuit Nunangat (Inuvialuit, Nunavut, Nunavik, and Nunatsiavut), and Makivvik Corporation, the NRBHSS is currently developing and organizing the next health survey—*Qanuippitaa?* National Inuit Health Survey—planned for Nunavik in the fall of 2023 and winter of 2024. This survey will then be conducted every 5 years and will be entirely led by Inuit. The lessons learned, some of which are shared here, will help inform this work.

## Contributions to knowledge

What does this study add to existing knowledge?

- *Qanuilirpitaa?* 2017 was the third major health survey conducted in Nunavik. It provided a much-needed update of the current health status of the population, covering physical health, mental health, and community health, and their determinants.
- A co-construction and co-interpretation approach allowed Nunavimmiut to take ownership of the process, results, and ensuing communications.
- Sociocultural factors were addressed for the first time and many were identified as potential protective factors of health and well-being of Nunavimmiut.

What are the key implications for public health interventions, practice, or policy?

- Findings of the survey can be used in advocacy efforts toward structural policy actions to improve income, food security, and housing conditions; the health and well-being of Nunavimmiut would improve as a result.
- Conducting health surveys in communities, spending more time in each of them, and restricting the number of themes addressed would improve the participation rate and the quality of the results.
- Providing adequate psychological support to participants and interviewers is an absolute requirement for future health surveys in the region.

### Supplementary Information

Below is the link to the electronic supplementary material.

Supplementary file1 (DOCX 167 KB)

## Data Availability

Not applicable.
