# Supplementary material for: The Qanuilirpitaa? 2017 Nunavik Health Survey: design, methods, and lessons learned
Source: Can J Public Health. 2024 Jan 17;115(Suppl 1):7–19. doi: 10.17269/s41997-023-00846-6 (PMC10830945; doi:10.17269/s41997-023-00846-6)

**Supplementary Material**

**Table S1 Blocks and sections of the *Qanuilirpitaa*? 2017 Health Survey questionnaire**

| **Block** | **Section** | **Reference of corresponding thematic reports** |
| --- | --- | --- |
| 1. Psychosocial interview (part I) | 1. Identity and spirituality | Sociocultural Determinants of Health and Wellness |
|  | 2. Well-being and support | Mental Health and Wellness Sociocultural Determinants of Health and Wellness |
|  | 3. Substance use and gambling | Substance Use  Gambling, Internet and Media Use |
|  | 4. Family | Sociocultural Determinants of Health and Wellness |
| 2. Physical health and food security interview | 1. Self-rated health | Mental Health and Wellness |
|  | 2. Non-intentional injuries | Unintentional Injuries |
|  | 3. Respiratory health | Respiratory Health |
|  | 4. Oral health | Oral Health |
|  | 5. Zoonoses | Zoonoses and gastrointestinal diseases |
|  | 6. Gastro-intestinal illnesses | Zoonoses and gastrointestinal diseases |
|  | 7. Practice of traditional activities and gun and ammunition use | Hunting, Fishing, Gathering, Ammunition and Public Health Messaging  Environmental Contaminants: Metals |
|  | 8. Contaminants and risk communication/perception | Hunting, Fishing, Gathering, Ammunition and Public Health Messaging |
|  | 9. Food security | Food Security |
| 3 Psychosocial interview (part II) | 5. Victimization | Interpersonal Violence and Community Safety |
|  | 5.1 Adverse experiences during childhood | Interpersonal Violence and Community Safety |
|  | 5.2 Adverse experiences during adulthood | Interpersonal Violence and Community Safety |
|  | 5.3 Elder victimization | Interpersonal Violence and Community Safety |
|  | 5.4 Bullying | Interpersonal Violence and Community Safety |
|  | 5.5 Discrimination | Interpersonal Violence and Community Safety |
|  | 5.6 Community safety | Interpersonal Violence and Community Safety |
|  | 6. Men’s health | Men’s health: Perception of Gender Roles |
|  | 7. Reproductive health | Sexual and Reproductive Health |
|  | 8. Sexual health | Sexual and Reproductive Health |
|  | 9. Housing | Housing and Drinking Water |
| 4. Food frequency questionnaire |  | Country and Market Food Consumption and Nutritional Status  Environmental Contaminants: Metals  Environmental Contaminants: Persistent Organic Pollutants and Contaminants of Emerging Concern |
| 5. Socio-demographic interview |  | Sociodemographic characteristics |

Questionnaires can be found in the thematic reports cited in the table and in the appendix of the methodological report (Hamel et al., 2020)

**Table S2 List of laboratory analyses performed on biological samples from participants in the *Qanuilirpitaa*? 2017 Health Survey**

| **Biological sample** | **Laboratory analysis** | **Rationale for inclusion in 2017** | **Targeted cohorts*** |
| --- | --- | --- | --- |
| **Blood** | Complete blood count  Clinical biochemistry  Vitamins, micronutrients  Contaminants  Specific IgEs  Antibody titers (*Cryptosporidium sp, Trichinella sp, Toxoplasma gondii, H. pylori)*  Syphilis | Screening for anemia Allergies  Cardiovascular disease risk factors  Diabetes  Kidney and liver diseases  Nutritional deficiencies  Exposure to environmental contaminants  Allergies  Zoonosis  Other infections  Sexually transmitted and blood-borne infections (STBBI) screening | Youth and adults  Youth |
| **Urine** | Clinical biochemistry  Contaminants | Screening for kidney diseases  Nutritional deficiencies  Exposure to environmental contaminants | Youth and adults |
|  | Chlamydia and gonorrhea | STBBI screening | Youth – men and Youth -women having their period or pregnant |
| **Vaginal swab** | Chlamydia and gonorrhea | STBBI screening | Youth–women not having their period and not pregnant |
| **Stool sample** | *H. pylori* (antigen)  Presence of occult blood | Screening for active *H. pylori* infection  Colorectal cancer screening | Youth and adults  Adults (50+) |

* Youth corresponds to the 16 to 30 age group and adults to those aged 31 and older.

**Figure S1 Health Passport**


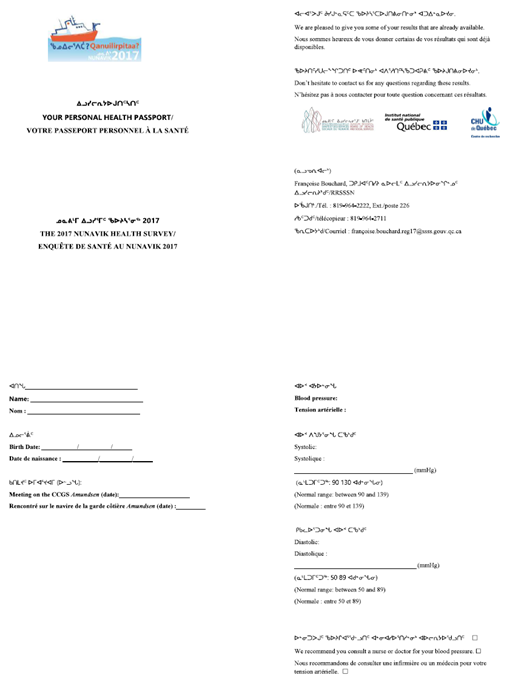


**Figure S1 Health Passport (continued)**


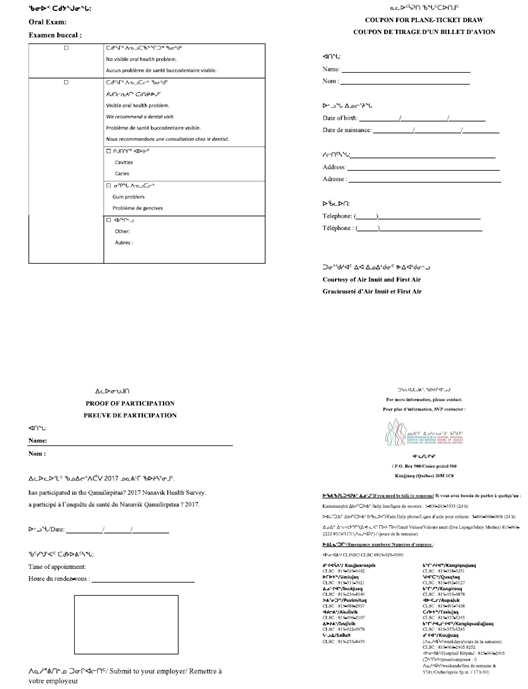

Supplement: Supplementary file 1 — Supplementary file1 (DOCX 167 KB) [file 41997_2023_846_MOESM1_ESM.docx]
